# Pitcher pot neourethral modification of ileal orthotopic neobladder achieves satisfactory long‐term functional and quality of life outcomes with low clean intermittent self‐catheterization rate

**DOI:** 10.1002/bco2.82

**Published:** 2021-06-04

**Authors:** Jiten Jaipuria, Ahmad Mamoon Karimi, Amitabh Singh, Bikash Bikram Thapa, Shashikant Gupta, Nripesh Sadasukhi, Manikandan Venkatasubramaniyan, Preeti Pathak, Priyatham Kasaraneni, Ashish Khanna, Tushar Aditya Narayan, Girish Sharma, Sudhir Rawal

**Affiliations:** ^1^ Amity Centre for Cancer Epidemiology & Cancer Research Amity Institute of Biotechnology Amity University Uttar Pradesh Noida India; ^2^ Department of Surgical Oncology Uro‐oncology Division Rajiv Gandhi Cancer Institute and Research Centre New Delhi India; ^3^ Centre for Medical Biotechnology, Coordinator Amity Centre for Cancer Epidemiology & Cancer Research Amity Institute of Biotechnology Amity University Uttar Pradesh Noida India

**Keywords:** neobladder, quality of life, radical cystectomy, robot‐assisted radical cystectomy, urinary bladder neoplasms, urinary diversion, urinary incontinence

## Abstract

**Objective:**

To describe a decade of our experience with a neo‐urethral modification of ileal orthotopic neobladder (pitcher pot ONB). Multiple investigators have reported similar modifications. However, long‐term longitudinal functional and quality of life (QOL) outcomes are lacking.

**Methods:**

Prospectively maintained hospital registry for 238 ONB patients comprising a mix of open and robotic surgery cohorts from 2007 to 2017, and minimum of 2 years of follow‐up was retrospectively queried. QOL was evaluated using Bladder Cancer Index (BCI). Longitudinal trends of QOL domain parameters were analysed. List of perioperative variables that have a biologically plausible association with continence, potency, and post‐operative BCI QOL sexual, urinary, and bowel domain scores was drawn. Variables included surgery type, Body Mass Index (BMI), T and N stage, neurovascular bundle (NVB) sparing, age, and related pre‐operative BCI QOL domain score. Prognostic associations were analysed using multivariable Cox proportional hazard models and multilevel mixed‐effects modeling.

**Results:**

The study comprised 80 and 158 patients who underwent open and robotic sandwich technique cohorts, respectively. Open surgery was associated with significantly higher “any” complication (40% vs 27%, *P*‐value .050) and “major” complication rate (15% vs 11%, *P*‐value .048). All patients developed a bladder capacity >400 cc with negligible post‐void residual urine, and all but one patient achieved spontaneous voiding by the end of study period (<1% clean intermittent self‐catheterization [CISC] rate). By 15 months, QOL for all three domains had recovered to reach a plateau. About 45% of patients achieved potency, and the median time to achieve day and night time continence was 9 and 12 months respectively. Lower age and NVBs spared during surgery were found to be significantly associated with the earlier achievement of potency, day and night time continence, as well as better urinary and sexual summary QOL scores.

**Conclusions:**

Pitcher pot neobladder achieves satisfactory long‐term functional and QOL outcomes with negligible CISC rate. Results were superior with incremental nerves spared during surgery.

## 1 INTRODUCTION

Bladder cancer has the second‐highest incidence rate among all genitourinary malignancies.^1^ Radical cystectomy remains the standard treatment for the muscle‐invasive disease. A systematic review by the ICUD–SIU International Consultation on Bladder Cancer concluded in favour of orthotopic neobladder (ONB) to have a better quality of life (QOL) in comparison to other forms of urinary diversion (UD) in the short and medium‐term.^2^ However, most studies analysing QOL were cross‐sectional, with scarce longitudinal data.

We preferred Studer neobladder (SN) in the initial years due to the simplicity of design.^3^ However, the inability to make the ONB reach the urethral stump in few patients prompted us to modify SN by creating a neourethra using a part of the ileum, thus aiding anastomosis without tension by providing extra length.^4^ The final appearance resembled an Indian earthenware container called a “pitcher pot.” The ease of neourethral anastomosis helped when we innovated the technique to reduce the length of surgical incision to 8‐12 cm (mini‐lap radical cystoprostatectomy).^5^ Ultimately, pitcher pot ONB became our default preference in every case irrespective of any consideration about the mesentery's length.

However, theoretical concerns existed about the possibility of an “accordion” or “concertina” effect whereby the neourethral tube could kink, leading to bladder outlet obstruction. Here we describe a decade of our experience with “pitcher pot” ONB, emphasising long‐term functional and QOL outcomes, including complications.

## 2 MATERIALS AND METHODS

Ours is a tertiary level regional cancer center, and a prospective registry of all surgical cases is maintained. Treatment protocols, including indications and contraindications to ONB, follow the National Comprehensive Cancer Network (NCCN) guidelines.^6^ We do not fashion neobladder in those with locally advanced tumours and restrict neoadjuvant chemotherapy to those with suspected T_3_ or N1 disease on pre‐operative imaging. Adjuvant chemotherapy was given to those with ≥T_3_ or node‐positive disease on final histopathology. This helps avoid overtreating patients having T2N0 disease, where the benefit of systemic chemotherapy is marginal. Supplementary Figure A summarizes the key steps to configure a pitcher pot ONB and video of the technique can be accessed from https://youtu.be/j7ZcltdJK5o.

Since 2011 performing cystectomy robotically became our preferred approach unless the patient picked open mini‐lap radical cystoprostatectomy surgery due to cost constraints. The “sandwich technique” for fashioning ONB was performed, where cystectomy was performed robotically, and the specimen was delivered via a small periumbilical incision which was also used to fashion the ONB extracorporeally. The robot was docked to complete the vesicourethral anastomosis. Recently, we have even begun to fashion this neobladder completely intracorporeally; however, a detailed description of the technique and outcomes is part of a separate manuscript.

### 2.1 Follow‐up schedule

The patient was discharged with maintenance antibiotics for 30 days. Following suture removal and review of histopathology to assess the need for any adjuvant chemotherapy on the 14 day, patients were instructed to report initially after 6 weeks of surgery. Subsequent visit schedule was once every 3 months for the first 2 years; every 6 months for the next 3 years; and then annually once afterwards. Imaging and laboratory workup for any metabolic abnormalities at each visit followed NCCN guidelines. For the first 2 years, complete blood count, a comprehensive metabolic panel with liver and kidney function tests (including electrolytes), and blood gas analysis were performed every 3‐6 months. In the years 2 through 5, the frequency of tests was reduced to once every 6 months, and vitamin B12 level was assessed in addition. After year 5, the frequency of tests was reduced to once per year. Routine urodynamic studies were not performed; however, all patients were taught positive life style changes such as pelvic floor exercises, drinking habits, maintaining healthy weight, renewed instructions on voiding techniques, and time intervals (especially at night time using an alarm clock). Any recorded complication was graded by Clavien Dindo classification; further dichotomized as major (≥3a) or minor (<3a).^7^ QOL was assessed at each visit using Bladder Cancer Index (BCI), which is a disease‐specific responsive instrument applicable to the entire spectrum of localized bladder cancer.^8^ It was initially described in 2007 and comprehensively summarized “urinary,” “bowel,” and “sexual” health domains of QOL via 36 items subdividing each domain into “function” and “bother” subdomains. The final score of each main domain or subdomain ranges from 0 to 100, with a higher score implying better QOL. Patients self‐administered the questionnaire at each visit (including first pre‐operative visit). In case of difficulty with language or interpretation, they were aided by a trained nurse practitioner with English as a native language.

With institutional ethics committee approval, we retrospectively collected data from prospectively maintained surgical registry for all male patients with localized bladder cancer who underwent ONB from June 2007 to June 2017 and had minimum of 2 years of follow‐up. The following outcome variables were analysed: serum creatinine, bladder capacity, post‐void residual urine (PVRU), uroflowmetry, day and night time continence, potency, and BCI QOL scores.

The patient was defined to be day and night time “continent” when he reported “total control” in response to items 26 and 27 of BCI, which assess “what response best describes your urinary leakage while awake” and “sleeping during past 4 weeks” respectively. The patient was defined to be “potent” if his response to item 56 of BCI assessing “ability to function sexually during the last 4 weeks” was at least “fair.”

We aimed to determine secular longitudinal trends of outcome parameters and uncover prognostic associations between them and biologically plausible perioperative variables.

## 3 STATISTICAL METHODS

Quantitative data with non‐parametric distribution are presented as median (interquartile range [IQR]), whereas standardized BCI scores are presented as mean (standard deviation [SD]). Count data are summarized as numbers (proportion).

For multivariable analysis, a list of perioperative variables having a biologically plausible association with continence, potency, and post‐operative BCI QOL domains was drawn including type of surgery, Body Mass Index (BMI), T and N stage, neurovascular bundle (NVB) sparing status, age, and related pre‐operative BCI QOL domain score. Charlson Comorbidity Index score was omitted as ONB was not offered to patients with significant comorbidities, and age remained the only factor contributing to higher scores, which was already accounted for.

### 3.1 Analysis of potency and continence

Cox proportional hazard models were used for multivariable analysis of the achievement of day and night time continence. Firth's penalised maximum likelihood survival analysis was performed for multivariable analysis of potency as a dependent outcome to overcome the problem of non‐convergence of maximum likelihood estimate.^9^

### 3.2 Analysis of BCI QOL domains

Two‐level (time as level 1, patients as level 2) random intercept mixed effect (maximum likelihood random‐effects type) multivariable model with each BCI QOL domain as the dependent outcome was created, with the same set of perioperative factors as independent variables (treated as fixed effects) as mentioned previously.

The entire data set was used for analysing secular trends of BCI QOL domain scores, serum creatinine, bladder capacity, and PVRU. For multivariable and multilevel analysis, we restricted the dataset to the first 2 years of follow‐up as outcomes plateaued beyond that time. It was almost universal to see patients miss some scheduled follow‐up visits over the extended study duration. Such events were mostly noted after the second year of follow‐up and were considered “missing at random” during the first 2 years for analysis. Descriptive statistics were analysed using MedCalc v15.8. Mann–Whitney U‐test was used for comparison of quantitative data, except for normally distributed BCI QOL domain scores which were analysed using t‐test (with Welch correction assuming unequal variances). Between‐group comparison for count data was made using Chi‐square or Fisher's exact test (if columns had less than five patients) as appropriate. Stata SE v14.2 was used for multilevel modeling; remainder analysis, including graphing, was done using the R program (v3.6.1). *α* < .05 was set as significant before‐hand.

## 4 RESULTS

### 4.1 Baseline and perioperative characteristics

Table 1 summarizes the demographic and perioperative details of the study population (n = 238). The study included 80 and 158 patients who underwent open and robotic surgery, respectively. Both groups were comparable in baseline demographic and clinical characteristics, including pre‐operative QOL scores. The majority of patients were pT2N0 and reported satisfactory pre‐operative urinary function and sexual bother scores. Patients undergoing robotic surgery had significantly higher median operative duration (352 vs 295 min, *P* value < .0001). However, the robotic cohort had lower median blood loss (220 vs 310 mL, *P* value < .0001) and hospital stay (eight vs nine days, *P* value < .0001). Median overall survival was not reached in either cohort; however, as robotic surgery was introduced later, follow‐up duration was significantly higher for open surgery group (102 vs 49 months, *P* value < .0001).

**TABLE 1:** Demographic and perioperative clinical details of entire study population stratified by type of surgical approach (n = 238)

Clinical parameters	Open approach n = 80	Robotic sandwich approach n = 158	*P* value
Age, years, median (IQR)	56 (54‐60)	58 (54‐61)	.17
Body Mass Index, median (IQR)	26 (24.9‐27)	25.8 (24.6‐26.9)	.63
CCI score, median (IQR)	3 (3–4)	3 (3–4)	.25
Preoperative serum creatinine, mg/dL, median (IQR)	0.8 (0.7–0.9)	0.8 (0.7–1)	.29
Preoperative BCI QOL domain and subdomain scores, mean (SD)			
Urinary summary	81.2 (6.6)	83.1 (5.1)	***.026***
Urinary function	100	100	NA
Urinary bother	71.9 (9.9)	74.7 (7.6)	***.026***
Bowel summary	91.1 (6)	90.1 (6.3)	.27
Bowel function	89 (7.6)	88.4 (7.5)	.52
Bowel bother	92.4 (8.7)	91.3 (9.2)	.36
Sexual summary	89.9 (7.7)	88.5 (8.8)	.23
Sexual function	82.7 (13.1)	80.3 (15.1)	.23
Sexual bother	100	100	NA
Pathological T stage, n			
T_1_	3	8	.85
T_2_	68	130	
T_3_	9	20	
Pathological N stage, n			
N_0_	79	150	.28
N_1_	1	8	
NVB spared, n			
None	7	20	.30
Unilateral	41	83	
Both	32	55	
Operative time, minutes, median (IQR)	295 (279‐307)	352 (330‐369)	***<.0001***
Blood loss, mL, median (IQR)	310 (300‐330)	220 (220‐230)	***<.0001***
Hospital stay, days, median (IQR)	9 (8‐10)	8 (7‐9)	***<.0001***
Follow up, months, median (IQR)	102 (84‐118)	49 (38‐67)	***<.0001***
Median OS	Not reached	Not reached	NA
Perioperative chemotherapy, n	9	20	.92

Note

*P* values rounded off to two significant decimals, significant *P* values marked **bold** and ***italicized***

Abbreviations: BCI QOL, bladder cancer index quality of life; CCI, Charlson's comorbidity index; IQR, interquartile range; NA, not applicable; NVB, neurovascular bundle; OS, overall survival.

### 4.2 BCI QOL domain trends

**FIGURE 1:**
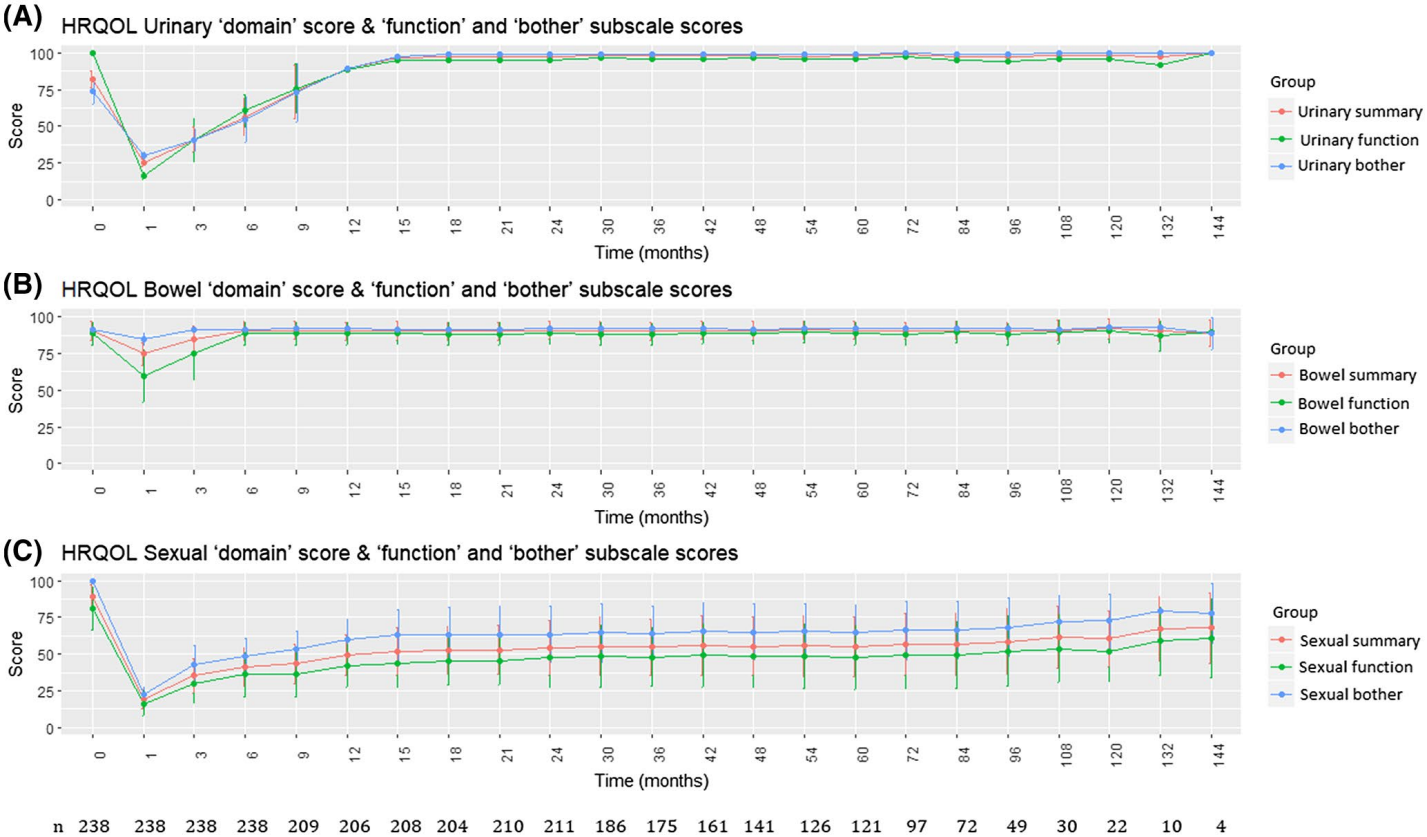
Line and error bar plot showing longitudinal trends of individual urinary, bowel, and sexual health domains of BCI for the entire study population

Figure 1 depicts secular longitudinal trends of individual urinary, bowel, and sexual health domains of BCI averaged for the entire study population, along with the number of patients enrolled at each follow‐up visit. Patients reported a sharp decline in QOL for all three domains after 6 weeks of surgery. The decrease was the smallest for bowel health‐related QOL, which was also first to recover. Urinary and sexual health‐related QOL showed gradual improvement up to 15 months, beyond which they plateaued. However, while urinary health‐related QOL reached levels similar to pre‐operative values for almost all patients; sexual health‐related QOL remained persistently low for many, resulting in lower overall domain scores.

### 4.3 Potency, urinary continence, and reservoir functional outcomes

Supplementary Figure B depicts the cumulative probability curves for achieving potency, daytime and night time continence for the entire study cohort restricting the sample to the first 24 months (as recovery beyond this time frame was rare). For the overall group, the median time to achieve day and night time continence was 9 (95%CI 9‐12) and 12 (95%CI 12‐15) months respectively. Four (<2%) patients did not achieve continence, and 130 (55%) patients didn't achieve potency.

Supplementary Table A summarizes the output from multivariate Cox proportional hazard survival analysis. Lower age and incremental NVBs spared during surgery were found significantly associated with the earlier achievement of potency, day as well as night time continence. The higher pathological T stage had an inverse association with the achievement of day and night time continence. Also, higher pre‐operative BCI sexual domain score was found to have a significant association with the achievement of potency postoperatively (HR 1.5 [95%CI 1.3‐1.8], *P* value < .0001).

Supplementary figure C shows longitudinal trends for bladder capacity, PVR urine (measured via ultrasound), and serum creatinine for the entire study cohort. Bladder capacity rose linearly over 2 years before reaching a plateau such that all patients achieved a bladder capacity > 400 mL, whereas PVRU averaged < 50 mL over the entire follow‐up period. Serum creatinine showed a gradual rise in the initial 2 years before plateauing for the next 3 years. Beyond the fifth year, it showed a secondary increase but stayed < 2mg/dL for most patients over a decade of follow up. Six patients needed revision for ureter‐enteric anastomotic stricture. However, this complication may not be ascribed to the ONB technique. Only one patient developed the need for renal replacement therapy. The mean uroflow rate was 11.8 (3.3) and 12.2 (3.3) mL/sec at the end of the first and second year, respectively.

### 4.4 Multivariable mixed effect multilevel models for QOL outcomes

Supplementary Table B summarizes the output from two‐level random intercept and random effect multivariate models with each BCI QOL domain as a dependent outcome. All three domain scores were found to be significantly associated with time and inversely associated with age. NVB sparing was found to be significantly associated with better urinary and sexual summary scores. A significant association was also noted between pre‐operative bowel and sexual summary scores with corresponding post‐operative QOL domain outcome. Supplementary figure D depicts the longitudinal trends for urinary and sexual BCI QOL domain summary scores stratified by variables found significant in multilevel mixed‐effects multivariable models.

### 4.5 Complications

Supplementary Table C summarizes the complications stratified by the surgical approach. One patient experienced perioperative mortality secondary to intestinal obstruction and aspiration pneumonia. Open surgery was associated with significantly higher “any” complication (40% vs 27%, *P*‐value .050) and “major” complication rate (15% vs 11%, *P*‐value .048). Following initial catheter removal, all but three patients voided spontaneously per urethra. All three voided spontaneously following the second trial void after 1 week and no patient required clean intermittent self‐catheterization (CISC) to empty the bladder. However, six patients developed bladder outlet obstruction secondary to hypertrophied mucosal folds obstructing the neourethra within 2 years. All responded well to endoscopic mucosal fold resection and resumed nonobstructed spontaneous voiding. Three patients needed another mucosal fold resection procedure. There was no kinking of the neourethral tube on the micturating cystourethrogram for all six patients. Three patients developed neo urethral anastomotic stricture, whereas two responded well to endoscopic dilatation, one developed the need for long‐term CISC.

## 5 DISCUSSION

To the best of our knowledge, this is the most extensive study to comprehensively assess long‐term rehabilitation of a patient with ONB with neo urethral modification in terms of longitudinal QOL assessments with a validated disease‐specific questionnaire. Most patients in our series achieved urinary domain summary scores similar to pre‐operative levels. However, potency sufficient for at least fair sexual performance was only achieved in 45% of patients. We found age, NVB‐spared status, and pre‐operative sexual summary scores as significant prognostic factors for potency. NVB sparing was also associated with the earlier return of day time urinary continence and had the highest effect size on multivariate analysis independent of the radicality of surgery dictated by the T stage, results consistent with those previously reported by Kessler et al in a series of 381 patients with SN.^10^ Night time continence recovers later, and we again found age and NVB sparing to be significantly associated with it. It is hypothesized that with age, urethral closing pressure and urethral innervation decrease. This may impact both voluntary and involuntary sphincter contractions and impair continence further potentiated by lack of neurofeedback to brain, increased night time diuresis, and osmotic shift of water into concentrated urine within the reservoir. As innervation plays a crucial role it is not surprising that others have also found any degree of nerve sparing to be associated with improved night time continence, which improves further with time.^11^ Better functional outcomes translate to better urinary and sexual domain summary QOL scores.

### 5.1 Maneuvres to resolve short mesentery and experience of others with neourethral modification

Short mesentery is an occasional concern needing surgeons’ attention while forming orthotopic neobladder. Some maneuvres to resolve it are common knowledge such as reducing the Trendelenburg tilt (during robotic surgery) and deeper incision in the distal mesentery of the isolated bowel limb.^12^ Further options include step ladder peritoneal cuts drawing from the experience of gastrointestinal surgeons.^13^ We described the creation of a neo‐urethra to resolve the problem of shortened mesentery and tension on vesicourethral anastomosis in 2006. Multiple investigators have subsequently explored the same concept.^14^ Rodolfo et al. reported experience with shortened mesentery in five patients due to previous abdominal surgeries and fashioned neourethra similar to ours to overcome anastomotic tension (though the reservoir was not spherical).^15^ Functional outcomes at one year were excellent, with all patients voiding spontaneously. They notably remarked about the ease of technique. Chandra et al. modified the Padua neobladder to construct an offset neourethral opening, which “facilitated ease in reaching the native urethra (even in obese individuals) and made a dependent funnel that promotes better bladder emptying.^16^ The bladder neck contracture rate was 2% in their series of 160 patients. Giampaolo et al. modified the SN to create a conical distal part that eased the urethroneovesical anastomosis and reduced anastomotic tension, which they hypothesized reduced the anastomotic stricture rate to 2.7% in their series of 36 patients.^17^

Despite all efforts, if mesentery still does not allow an ileal neobladder then sigmoid neobladder can be considered which may provide a better chance at spontaneous voiding, though at the cost of lower patient satisfaction.^18^

### 5.2 Voiding

Though the initial drive to innovate pitcher pot neobladder was shortened mesentery, later it became out default choice in every case because it eased the vesico‐neourethral anastomosis (particularly advantageous in robotic surgery where patients are in Trendelenburg position, and manipulative abilities are limited), and facilitated excellent voiding. All patients voided spontaneously in our series following catheter removal with reasonable uroflow rates and low PVRU, implying excellent functional reservoir dynamics. Explaining CISC has ceased to be a part of our routine care protocols since over a decade now. Similar excellent spontaneous voiding rates have been described with all neourethra type modifications of the neobladder. However, an 8.8% dysfunctional voiding rate (independent of physical obstruction to bladder outlet) necessitating CISC has been described by others for SN.^19^ A recent series has reported a CISC rate of 22% with SN necessitated by “bladder overdistension, deteriorating renal function, or recurrent urosepsis despite timed voiding”.^20^ Nerve‐sparing and younger age have been found associated with improved CISC rates.^21^ Negligible CISC rate might also have contributed to high observed post‐operative QOL summary scores. Studer et al. noted kinking of bladder outlet due to its funnel shape leading to obstruction in four patients though others have hypothesized the reverse.^16^, ^22^ “Dependent funnel that promotes better bladder emptying” and “creating the neourethra in the centre‐lowermost part of the reservoir rather than one extreme of the suture line”' might explain why we and others have observed excellent voiding function and not encountered a problem with kinking of the bladder outlet or neourethra. Lower tension at the anastomosis and better inherent design of suturing a tube (neourethra) to tube (native urethral stump) with a higher theoretical probability of perfect mucosa to mucosa coaptation may explain the low vesicourethral anastomotic stricture rate of 1.2% in our series. In comparison, it has been reported to be as high as 16.9% in the literature.^17^, ^22^ Atleast 2.1% of patients in our series developed obstructing neourethral mucosal folds during follow up, which responded well to endoscopic resection. Such obstructing mucosal folds were also noted by Studer in 34 patients in an extensive follow‐up series of 482 patients with SN.^23^ Resecting such folds are safe, and the surgeon should limit oneself to resecting the mucosal layer only, aiming to achieve an unobstructed passage. With due diligence, spontaneous voiding can be achieved, and bladder perforation is not yet reported.^23^, ^24^

It is essential to understand that pitcher pot modification does not need an additional length of the bowel to be sacrificed, but only requires central 5 cm portion to be fashioned into a neourethral stump. Hence, it does not predispose patients to any higher risk of metabolic dysfunction than the conventional SN.

We observed a lower “major” and “any” complication rate with robotic surgery, and all neobladders were fashioned via a sandwich approach. Literature comparing both approaches’ complications is controversial; however, a recent review noted a trend toward lower complications with robotic surgery.^25^ However, the robotic platform does allow higher magnification and improved maneuvring in deeper areas of the pelvis. Unsurprisingly, some investigators have noted a higher median lymph node yield with robotic than open surgery.^25^, ^26^, ^27^ Atmaca et al. also noted better NVB sparing with the robotic approach with resultant better daytime continence rate.^27^ It remains conjectural if robotic surgery also allows a better urethral anastomosis.

### 5.3 Limitations

We have not directly compared pitcher pot with SN and, thus, our results should not be concluded to be superior by indirect comparison of historical data. Further, patients in our cohort are relatively younger though we equally offered ONB to all patients less than 70 years of age. BCI has to be self‐administered, but many patients were not native English speakers. We tried to reduce the interviewer bias by ensuring that an independent trained nurse practitioner specialising in rehabilitating patients with urinary diversions and post‐prostatectomy urinary incontinence administered the questionnaire. They regularly administer other questionnaires such as IPSS and cancer‐specific QOL scores such as FACT and EORTC for which vernacular translated versions are available. There is no reason to believe that sufficient explanation was not given to every patient.

While we assessed functional bladder capacity by USG and PVRU, other aspects such as objective voiding pressures would need a urodynamic study. We reported satisfactory urodynamic parameters with the original description of the pitcher pot ONB in 21 patients in the year 2006, and subsequently stopped performing it due to lack of clinical utility. Good urodynamics should ultimately translate to adequate renal reserves and urinary QOL scores, and both have been addressed in the manuscript. Further, we did not rely on pad usage to assess continence as BCI has specific questions focusing on this issue. There is no reason to assume incontinence if the patient replies as “total control” regarding urinary leakage during daytime and sleeping.

Lastly, ours is a private cancer hospital and, as such, almost all our patients are socioeconomically advanced, at least to a certain degree. Open surgery cohort overwhelmingly comprised of patients before 2011 when our first robotic console was installed. A patient preferred open surgery in the later years only occasionally (primarily driven by cost concerns). We have not captured socioeconomic data according to a validated scale; however, analysing this aspect should not have a significant bearing on the primary area under the manuscript's focus.

## 6 CONCLUSION

Pitcher pot ONB achieves satisfactory long‐term functional outcomes with negligible CISC rate. Patients achieved urinary domain QOL scores similar to pre‐operative levels by the end of one year. Sparing of NVBs was found to be the most substantial modifiable factor associated with improved post‐operative urinary and sexual domain QOL, as well as the earlier return of potency, day, and night time continence.

## AUTHOR CONTRIBUTIONS

J Jaipuria performed project development, data collection, data analysis, and manuscript writing/editing. MA Karimi performed data collection, data analysis, and manuscript writing. A Singh carried out project development, data collection, data analysis, and manuscript editing. BB Thapa performed data collection and manuscript writing. S Gupta performed data collection and manuscript writing. N Sadasukhi—data collection, manuscript writing. M Venkatasubramaniyan performed data collection, data analysis, manuscript writing. P Pathak performed data collection and manuscript writing. P Kasaraneni performed data collection and manuscript writing. A Khanna performed data collection and manuscript writing. TA Narayan performed data collection and manuscript writing. GS performed data analysis, manuscript writing, and manuscript editing. S Rawal performed project development, data collection, and manuscript editing.

## CONFLICT OF INTEREST

The authors declare that they have no conflict of interest.

## INFORMED CONSENT

Informed consent was obtained from all individual participants included in the study.

## RESEARCH INVOLVING HUMAN PARTICIPANTS AND/OR ANIMALS

Ethical approval was waived by the local Ethics Committee of the Institute in view of the retrospective nature of the study and all the procedures being performed were part of the routine care.

## References

[1] Bray F , Ferlay J , Soerjomataram I , Siegel RL , Torre LA , Jemal A . Global cancer statistics 2018: GLOBOCAN estimates of incidence and mortality worldwide for 36 cancers in 185 countries. CA Cancer J Clin. 2018;68:394–424. Erratum in: CA Cancer J Clin. 2020;70:313. 30207593 10.3322/caac.21492

[2] Faba OR , Tyson MD , Artibani W , Bochner BH , Burkhard F , Gilbert SM . Update of the ICUD‐SIU International Consultation on Bladder Cancer 2018: urinary diversion. World J Urol. 2019;37:85–93. 30238399 10.1007/s00345-018-2484-3

[3] Studer UE , Varol C , Danuser H . Surgical atlas orthotopic ileal neobladder. BJU Int. 2004;93:183–93. 14678400 10.1111/j.1464-410x.2004.04641.x

[4] Rawal S , Kumar P , Kaul R , Raghunath SK , Julka S . The 'pitcher pot' ileal neobladder: early experiences. Jpn J Clin Oncol. 2006;36:717–22. 17003076 10.1093/jjco/hyl100

[5] Rawal S , Raghunath SK , Khanna S , Jain D , Kaul R , Kumar P , et al. Minilaparotomy Radical Cystoprostatectomy (Minilap RCP) in the surgical management of urinary bladder carcinoma: early experience. Jpn J Clin Oncol. 2008;38:611–6. 18772171 10.1093/jjco/hyn079

[6] NCCN guidelines: Bladder cancer . [cited 2019 Oct 19]. Available from: https://www.nccn.org/professionals/physician_gls/pdf/bladder.pdf

[7] Dindo D , Demartines N , Clavien PA . Classification of surgical complications: a new proposal with evaluation in a cohort of 6336 patients and results of a survey. Ann Surg. 2004;240:205–13. 15273542 10.1097/01.sla.0000133083.54934.ae PMC1360123

[8] Gilbert SM , Wood DP , Dunn RL , Weizer AZ , Lee CT , Montie JE , et al. Measuring health‐related quality of life outcomes in bladder cancer patients using the Bladder Cancer Index (BCI). Cancer. 2007;109:1756–62. 17366596 10.1002/cncr.22556

[9] Firth D . Bias reduction of maximum likelihood estimates. Biometrika. 1993;80:27–38.

[10] Kessler TM , Burkhard FC , Perimenis P , Danuser H , Thalmann GN , Hochreiter WW , et al. Attempted nerve sparing surgery and age have a significant effect on urinary continence and erectile function after radical cystoprostatectomy and ileal orthotopic bladder substitution. J Urol. 2004;172:1323–7. 15371833 10.1097/01.ju.0000138249.31644.ec

[11] Furrer MA , Studer UE , Gross T , Burkhard FC , Thalmann GN , Nguyen DP . Nerve‐sparing radical cystectomy has a beneficial impact on urinary continence after orthotopic bladder substitution, which becomes even more apparent over time. BJU Int. 2018;121:935–44. 29319917 10.1111/bju.14123

[12] Chesnut GT , Rentea RM , Leslie SW . Urinary diversions and neobladders. [Updated 2020 Nov 12]. In: StatPearls [Internet]. Treasure Island, FL: StatPearls Publishing; [cited 2020 Jan]. Available from: https://www.ncbi.nlm.nih.gov/books/NBK560483/ 32809318

[13] Baig MK , Weiss EG , Nogueras JJ , Wexner SD . Lengthening of small bowel mesentery: stepladder incision technique. Am J Surg. 2006;191:715–7. 16647367 10.1016/j.amjsurg.2005.08.032

[14] Hautmann RE . Ileal neobladder. BJU Int. 2010;105:1024–35. 20356328 10.1111/j.1464-410X.2010.09283.x

[15] Dos Reis RB , Machado RD , Faria EF , Cassini M , Kaplan S . Modified technique for the creation of an orthotopic neobladder in patients with shortened mesentery: making up the difference between the bladder and the urethral stump. Urology. 2011;78:1430–4. 21996104 10.1016/j.urology.2011.07.1404

[16] Flack CK , Monn MF , Kaimakliotis HZ , Koch MO . Functional and clinicopathologic outcomes using a modified vescicailealepadovana technique. Bladder Cancer. 2015;1:73–9. 30561444 10.3233/BLC-140002 PMC6218179

[17] Bianchi G , Sighinolfi MC , Pirola GM , Micali S . Studer orthotopic neobladder: a modified surgical technique. Urology. 2016;88:222–5. 26627374 10.1016/j.urology.2015.11.020

[18] Tao S , Long Z , Zhang XJ , Zhu D , Shi XJ , Tan WL , et al. Ileal versus sigmoid neobladder as bladder substitute after radical cystectomy for bladder cancer: a meta‐analysis. Int J Surg. 2016;27:39–45. 26804352 10.1016/j.ijsu.2016.01.044

[19] Tanaka T , Kitamura H , Takahashi A , Masumori N , Itoh N , Tsukamoto T . Long‐term functional outcome and late complications of Studer's ileal neobladder. Jpn J Clin Oncol. 2005;35:391–4. 15976064 10.1093/jjco/hyi112

[20] Chan EP , Nair SM , Hetou K , Stephenson E , Power NE , Izawa J , et al. Longitudinal experience with Studer neobladders: outcomes and complications. Can Urol Assoc J. 2021;15(8). 10.5489/cuaj.6893 PMC8418254 33410740

[21] Murray KS , Arther AR , Zuk KP , Lee EK , Lopez‐Corona E , Holzbeierlein JM . Can we predict the need for clean intermittent catheterisation after orthotopic neobladder construction? Indian J Urol. 2015;31:333–8. 26604445 10.4103/0970-1591.166460 PMC4626918

[22] Nieuwenhuijzen JA , de Vries RR , Bex A , van der Poel HG , Meinhardt W , Antonini N , et al. Urinary diversions after cystectomy: the association of clinical factors, complications and functional results of four different diversions. Eur Urol. 2008;53:834–44. 17904276 10.1016/j.eururo.2007.09.008

[23] Studer UE , Burkhard FC , Schumacher M , Kessler TM , Thoeny H , Fleischmann A , et al. Twenty years experience with an ileal orthotopic low pressure bladder substitute–lessons to be learned. J Urol. 2006;176:161–6. 16753394 10.1016/S0022-5347(06)00573-8

[24] Schneider MP , Burkhard FC . Management of incontinence after orthotopic bladder substitution post‐radical cystectomy. Curr Bladder Dysfunct Rep. 2019;14:125–9.

[25] Elsayed AS , Aldhaam NA , Nitsche L , Siam A , Jing Z , Hussein AA , et al. Robot‐assisted radical cystectomy: review of surgical technique, and perioperative, oncological and functional outcomes. Int J Urol. 2020;27:194–205. 31981379 10.1111/iju.14178

[26] Xia L , Wang X , Xu T , Zhang X , Zhu Z , Qin L , et al. Robotic versus open radical cystectomy: an updated systematic review and meta‐analysis. PLoS One. 2015;10:e0121032. 25825873 10.1371/journal.pone.0121032 PMC4380496

[27] Atmaca AF , Canda AE , Gok B , Akbulut Z , Altinova S , Balbay MD . Open versus robotic radical cystectomy with intracorporeal Studer diversion. JSLS. 2015;19(1):e2014.00193. 10.4293/JSLS.2014.00193 PMC4376220 25848187

