# Supplementary figures and images for: Pitcher pot neourethral modification of ileal orthotopic neobladder achieves satisfactory long‐term functional and quality of life outcomes with low clean intermittent self‐catheterization rate
Source: BJUI Compass. 2021 Jun 4;2(4):292–9. doi: 10.1002/bco2.82 (PMC8988529; doi:10.1002/bco2.82)

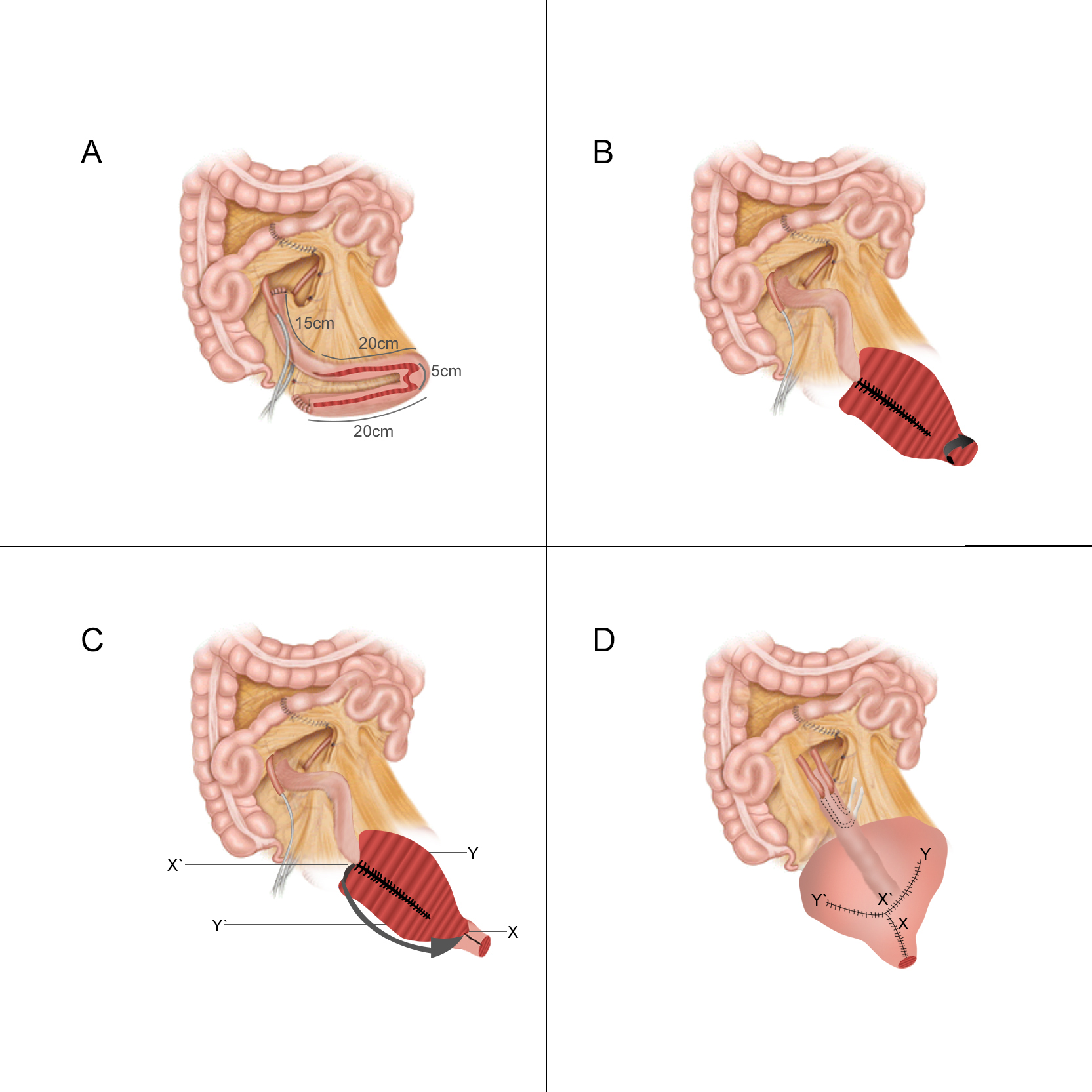

Supplement: Supplementary file 1 [file BCO2-2-292-s001.jpg]

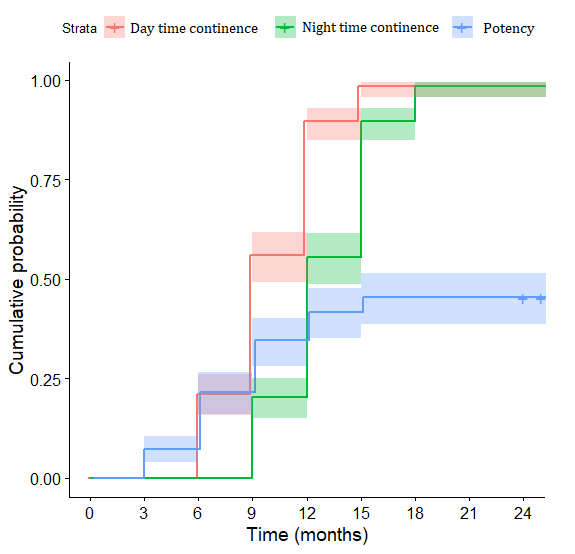

Supplement: Supplementary file 2 [file BCO2-2-292-s007.tif]

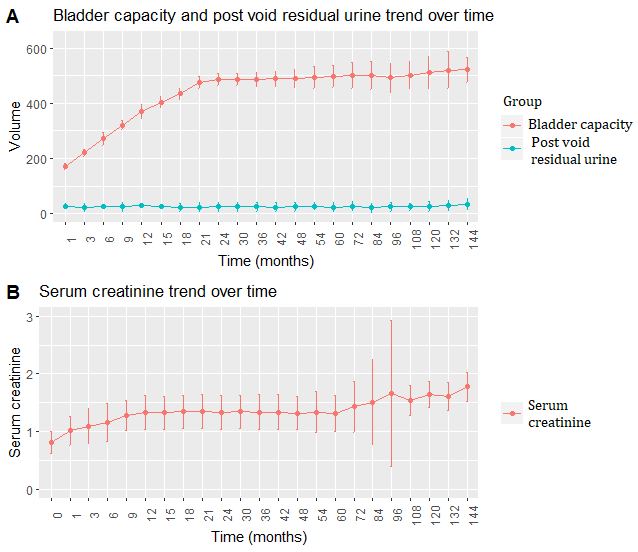

Supplement: Supplementary file 3 [file BCO2-2-292-s005.tif]

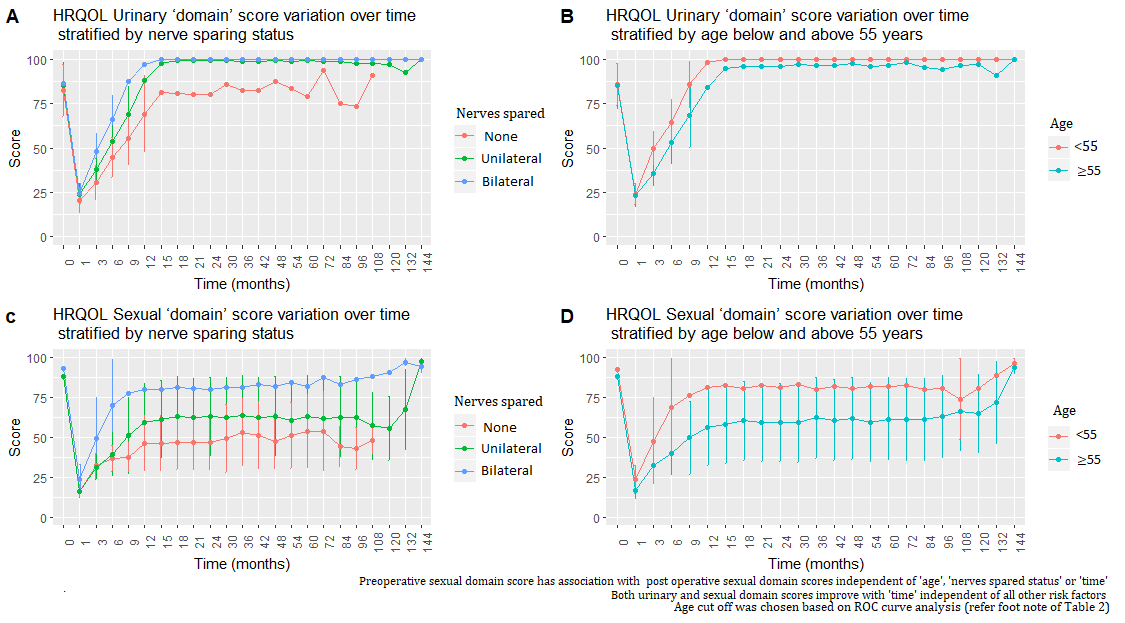

Supplement: Supplementary file 4 [file BCO2-2-292-s003.tif]
