# Supplementary material for: Pitcher pot neourethral modification of ileal orthotopic neobladder achieves satisfactory long‐term functional and quality of life outcomes with low clean intermittent self‐catheterization rate
Source: BJUI Compass. 2021 Jun 4;2(4):292–9. doi: 10.1002/bco2.82 (PMC8988529; doi:10.1002/bco2.82)
Supplement: Supplementary file 5 [file BCO2-2-292-s004.docx]

**Supplementary Table B:** Multivariable two-level (time and patients as levels 1 and 2 respectively) random intercept mixed effect (maximum likelihood random effect type) models with each BCI QOL domain as the dependent outcome.

| Variable | Urinary summary  Coefficient  (standard error),  p value | Bowel summary  Coefficient  (standard error),  p value | | | Sexual summary  Coefficient (standard error),  p value |
| --- | --- | --- | --- | --- | --- |
| Age | ***-0.39 (0.1), <0.0001*** | ***-0.06 (0.02), 0.013*** | | ***-1.6 (0.16), <0.0001*** | |
| Body Mass Index | -0.27 (0.23), 0.25 | 0.031 (0.06), 0.59 | | -0.47 (0.37), 0.21 | |
| Type of surgery^α^ | 1.4 (0.88), 0.12 | -0.024 (0.22), 0.91 | | 2 (1.4), 0.15 | |
| NVB spared^β^ |  | |  |  |  |
| Unilateral | ***9.9 (1.9), <0.0001*** | -0.15 (0.47), 0.74 | | 4 (3), 0.19 | |
| Bilateral | ***15.1 (2), <0.0001*** | -0.26 (0.49), 0.60 | | ***11.7 (3.2), <0.0001*** | |
| Pathological N_1_ stage^Ω^ | -1.2 (2.6), 0.64 | -0.087 (0.64), 0.89 | | 3.5 (4.1), 0.40 | |
| Pathological T stage^∞^ |  |  | |  | |
| T_2_ | 0.44 (2), 0.82 | -0.06 (0.5), 0.91 | | 2.4 (3.2), 0.45 | |
| T_3_ | -2.17 (2.9), 0.45 | -0.44 (0.7), 0.53 | | -4.2 (4.5), 0.36 | |
| Preoperative BCI QOL domain summary score^∑^ | -0.093 (0.08), 0.25 | ***1.02 (0.02), <0.0001*** | | ***1.3 (0.08), <0.0001*** | |
| Time* | ***9.6 (0.11), <0.0001*** | ***1.4 (0.04), <0.0001*** | | ***5.5 (0.14), <0.0001*** | |
| Constant | ***60.2 (11.1), <0.0001*** | ***-6.3 (2.7), 0.019*** | | 3.8 (16.4), 0.82 | |
| NVB – neurovascular bundle, BCI QOL – Bladder Cancer Index Quality of Life  Odds ratios rounded off to two decimals in case <1, otherwise rounded off to one decimal, p values rounded off to two significant decimals  Significant results marked *bold and italicised*.  ^α^ Odds ratio for robotic surgery with open surgery as reference  ^β^ With no NVB spared as reference  ^Ω^ With N_0_ stage as reference  ^∞^ With T_1_ stage as reference  ^∑^ BCI Urinary, bowel and sexual summary score for models with urinary, bowel and sexual BCI QOL as dependent outcome respectively  * Treated as continuous variable due to many time points. | | | | | |
