# Supplementary material for: Pitcher pot neourethral modification of ileal orthotopic neobladder achieves satisfactory long‐term functional and quality of life outcomes with low clean intermittent self‐catheterization rate
Source: BJUI Compass. 2021 Jun 4;2(4):292–9. doi: 10.1002/bco2.82 (PMC8988529; doi:10.1002/bco2.82)
Supplement: Supplementary file 6 [file BCO2-2-292-s006.docx]

**Supplementary Table A:** Multivariable cox proportional hazard survival analyses of risk factors associated with potency, day and night time continence.

| Variable | Daytime continence  Hazard ratio  (standard error),  p value | Night time continence  Hazard ratio  (standard error),  p value | | | Potency  Hazard ratio (standard error),  p value* |
| --- | --- | --- | --- | --- | --- |
| Age | ***0.95 (0.021), 0.0097*** | ***0.94 (0.022), 0.0038*** | | ***0.82 (0.03), <0.0001*** | |
| Body Mass Index | 0.98 (0.039), 0.57 | 0.98 (0.04), 0.67 | | 0.92 (0.06), 0.15 | |
| Type of surgery^α^ | 1.2 (0.14), 0.21 | 1.2 (0.14), 0.26 | | 0.92 (0.22), 0.69 | |
| NVB spared^β^ |  | |  |  |  |
| Unilateral | ***2.5 (0.31), 0.0029*** | ***2.4 (0.31), 0.0037*** | | ***7.9 (0.86), 0.025*** | |
| Bilateral | ***7.2 (0.33), <0.0001*** | ***6.5 (0.33), <0.0001*** | | ***11.6 (0.87), 0.0084*** | |
| Pathological N_1_ stage^Ω^ | 0.94 (0.45), 0.89 | 0.92 (0.45), 0.85 | | 1.1 (0.85), 0.93 | |
| Pathological T stage^∞^ |  |  | |  | |
| T_2_ | ***0.30 (0.32), 0.00024*** | ***0.54 (0.021), 0.055*** | | 1.4 (0.47), 0.44 | |
| T_3_ | ***0.39 (0.46), 0.040*** | 0.67 (0.021), 0.38 | | 1.9 (0.88), 0.48 | |
| Preoperative BCI QOL domain summary score^∑^ | 0.99 (0.01), 0.51 | 0.99 (0.01), 0.45 | | ***1.5 (0.07), <0.0001*** | |
| NVB – neurovascular bundle, BCI QOL – Bladder Cancer Index Quality of Life  Odds ratios rounded off to two decimals in case <1, otherwise rounded off to one decimal, p values rounded off to two significant decimals  Significant results marked *bold and italicised*  * Modelled using Firth’s penalised maximum likelihood cox proportional hazard model to overcome the problem of non-convergence of likelihood function.  ^α^ Odds ratio for robotic surgery with open surgery as reference  ^β^ With no NVB spared as reference  ^Ω^ With N_0_ stage as reference  ^∞^ With T_1_ stage as reference  ^∑^ BCI Urinary summary score for models with day and night time continence as dependent outcome. BCI Sexual summary score for model with potency as dependent outcome  To determine optimal cut off of association of a quantitative variable with the categorical outcome, receiver operating characteristic (ROC) curves were analyzed to determine the Youden index criterion (which maximizes balance of sensitivity and specificity). Youden index criteria of age for optimally discriminating those achieving day and night time continence was age <58 years (area under ROC curve 0.79, 59% sensitivity, 100% specificity for both outcomes), while that for potency was age <53 years (area under ROC curve 0.77, 44% sensitivity, 99% specificity). Pre-op BCI sexual summary score >85.4 was the Youden index criteria having 99% sensitivity and 78% specificity in discriminating those achieving potency postoperatively (area under ROC curve 0.94). | | | | | |
