# Supplementary material for: Pitcher pot neourethral modification of ileal orthotopic neobladder achieves satisfactory long‐term functional and quality of life outcomes with low clean intermittent self‐catheterization rate
Source: BJUI Compass. 2021 Jun 4;2(4):292–9. doi: 10.1002/bco2.82 (PMC8988529; doi:10.1002/bco2.82)
Supplement: Supplementary file 7 [file BCO2-2-292-s002.docx]

**Supplementary Table C:** Comparison of complications stratified by the type of surgical approach

| **Outcome** | **Open surgery**  **n=80** | **Robotic surgery**  **n=158** | **p-value** |
| --- | --- | --- | --- |
| **Complications** |  |  |  |
| Any complication, n (%) | 32/80 (40%) | 42/158 (27%) | ***0.050*** |
| Major complication^#^, n (%) | 12/80 (15%) | 17/158 (11%) | ***0.048*** |
| **Individual complications stratified by Clavien Dindo grade ^@^** | | | |
| **Grade 1, n** |  |  |  |
| Paralytic ileus | 9 | 15 |  |
| Lymphorrhea | 10 | 15 |  |
| Urine leak managed by prolonged Foley catheterization | 2 | 2 |  |
| Superficial surgical site infection | 7 | 6 |  |
| **Grade 2, n** |  |  |  |
| Deep surgical site infection | 3 | 4 |  |
| Blood loss needing transfusion | 4 | 4 |  |
| Pneumonia | 2 | 4 |  |
| Pyelonephritis | 5 | 7 |  |
| Paralytic ileus managed by therapeutic gastrograffin | 4 | 6 |  |
| Deep venous thrombosis | 2 | 3 |  |
| **Grade 3a, n** |  |  |  |
| Urinary leak requiring PCN | 1 | 1 |  |
| Lymphocele requiring drainage | 1 | 2 |  |
| Hydronephrosis requiring PCN | 4 | 5 |  |
| Pelvic hematoma requiring drainage | 1 | 1 |  |
| **Grade 3b, n** |  |  |  |
| Urinary leak needing operative exploration | 2 | 2 |  |
| Bowel obstruction needing exploration | 3 | 3 |  |
| Burst abdomen | 1 | 1 |  |
| Ureteroileal obstruction needing surgical revision | 3 | 3 |  |
| Neourethral stricture needing endoscopic management | 1 | 2 |  |
| Obstructing neourethral mucosal folds needing endoscopic resection | 3 | 3 |  |
| **Grade 4a, n** |  |  |  |
| Renal failure needing haemodialysis | 1 | 0 |  |
| ARDS | 1 | 1 |  |
| Acute coronary syndrome | 0 | 1 |  |
| **Grade 4b, n** |  |  |  |
| Sepsis with shock | 1 | 1 |  |
| Multiple organ failure | 0 | 1 |  |
| Severe metabolic acidosis with shock | 2 | 1 |  |
| **Grade 5, n** |  |  |  |
| Death | 1 | 1 |  |

PCN – percutaneous nephrostomy, ARDS – acute respiratory distress syndrome

* defined as those < Clavien 3a

^#^ defined as those ≥ Clavien 3a,

Between-group comparison for count data was done using Chi-square test

^@^ one patient could experience more than one complication event

Significant p values marked **bold** and ***italicized***
